# *Smad4* Loss Synergizes with *TGFα* Overexpression in Promoting Pancreatic Metaplasia, PanIN Development, and Fibrosis

**DOI:** 10.1371/journal.pone.0120851

**Published:** 2015-03-24

**Authors:** Dario Garcia-Carracedo, Chih-Chieh Yu, Nathan Akhavan, Stuart A. Fine, Frank Schönleben, Naoki Maehara, Dillon C. Karg, Chuangao Xie, Wanglong Qiu, Robert L. Fine, Helen E. Remotti, Gloria H. Su

**Affiliations:** 1 The Department of Pathology, Columbia University Medical Center, New York, New York, United States of America; 2 Herbert Irving Comprehensive Cancer Center, Columbia University Medical Center, New York, New York, United States of America; 3 The Department of Vascular Surgery in the Hospital of the University of Munich, Grosshadern, Germany; 4 Department of Surgical Oncology and Regulation of Organ Function, Miyazaki University School of Medicine, Miyazaki, Japan; 5 Department of Medicine, Columbia University Medical Center, New York, New York, United States of America; 6 Pancreas Center, Columbia University Medical Center, New York, New York, United States of America; 7 Department of Otolaryngology and Head and Neck Surgery, Columbia University Medical Center, New York, New York, United States of America; Garvan Institute of Medical Research, AUSTRALIA

## Abstract

**Aims:**

While overexpression of *TGFα* has been reported in human pancreatic ductal adenocarcinoma (PDAC), mice with overexpressed *TGFα* develop premalignant pancreatic acinar-to-ductal metaplasia (ADM) but not PDAC. TGF-β signaling pathway is pivotal to the development of PDAC and tissue fibrosis. Here we sought to investigate the interplay between TGFα and TGF-β signaling in pancreatic tumorigenesis and fibrosis, namely via *Smad4* inactivation.

**Methods:**

The *MT-TGFα* mouse was crossed with a new *Smad4* conditional knock-out mouse (*Smad4^flox/flox^;p48-Cre* or *S4*) to generate *Smad4^flox/flox^;MT-TGFα;p48-Cre (STP)*. After TGFα overexpression was induced with zinc sulfate water for eight months, the pancreata of the *STP*, *MT-TGFα*, and *S4* mice were examined for tumor development and fibrotic responses. PanIN lesions and number of ducts were counted, and proliferation was measured by Ki67 immunohistochemistry (IHC). Qualitative analysis of fibrosis was analyzed by Trichrome Masson and Sirius Red staining, while vimentin was used for quantification. Expression analyses of fibrosis, pancreatitis, or desmoplasia associated markers (*α-SMA*, *Shh*, *COX-2*, *Muc6*, *Col1a1*, and *Ctgf*) were performed by IHC and/or qRT-PCR.

**Results:**

Our *STP* mice exhibited advanced ADM, increased fibrosis, increased numbers of PanIN lesions, overexpression of chronic pancreatitis-related marker Muc6, and elevated expression of desmoplasia-associated marker Col1A1, compared to the *MT-TGFα* mice. The inactivation of *Smad4* in the exocrine compartment was responsible for both the enhanced PanIN formation and fibrosis in the pancreas. The phenotype of the *STP* mice represents a transient state from ADMs to PanINs, closely mimicking the interface area seen in human chronic pancreatitis associated with PDAC.

**Conclusion:**

We have documented a novel mouse model, the *STP* mice, which displayed histologic presentations reminiscent to those of human chronic pancreatitis with signs of early tumorigenesis. The *STP* mice could be a suitable animal model for interrogating the transition of chronic pancreatitis to pancreatic cancer.

## Introduction

Pancreatic ductal adenocarcinoma (PDAC) is the most common neoplasm of the pancreas [1]. Despite the low incidence of 6–12 per 100,000 per year, PDAC is the fourth leading cause of cancer deaths in the United States due to the lack of early detection methods and effective treatments [2]. While the cell of origin remains to be clearly defined, it is proposed that pancreatic cancer can progress from acinar to ductal metaplasia (ADM) and is a result of the combination of genetic events and extrinsic factors that produce tissue injury, such as the associated inflammatory damages observed during pancreatitis [3, 4]. Chronic pancreatitis is characterized by fibroinflammatory changes of the pancreatic tissue and has been shown to be a risk factor for pancreatic cancer [5]. Transforming growth factor-alpha (TGFα) is a member of the epidermal growth factor (EGF) family of cytokines, which acts in autocrine and paracrine fashions by binding to the EGF receptor to regulate cell proliferation, differentiation, transformation, and migration [6]. Overexpression of TGFα has been reported in transformed cells of many cancer types, including the acinar cells and ductal epithelium in human pancreatic cancer [7, 8]. Transgenic mice expressing TGFα transgene under the control of zinc-inducible metallothionein (MT) promoter/enhancer or elastase promoter exhibited progressive pancreatic fibrosis, loss of acinar cell mass, and development of extensive tubular complexes, termed pseudoductular metaplasia [9, 10].

*SMAD4*, originally isolated from human chromosome 18q21.1, is a key intracellular mediator of transcriptional responses to TGF-β. TGF-β plays a complicated, biphasic stage-specific role in tumorigenesis by serving as a tumor-suppressor during early initiation, and yet promoting tumor progression in late stages [11]. As a central effector of the TGF-β pathway, *SMAD4* is believed to be a tumor-suppressor gene as evidenced by being biallelically inactivated in more than 50% of pancreatic carcinomas [12]. *Smad4* deficiency can lead to rapid progression of pancreatic tumors in the context of activated *Kras* ^*G12D*^; however, *Smad4* deficiency alone is incapable of initiating pancreatic tumorigenesis and dispensable for normal pancreas development [13–15].

In addition to its importance in tumorigenesis, TGF-β signaling has long been recognized to induce extracellular matrix (ECM) synthesis and tissue fibrosis [16, 17]. While fibrotic disease represents a large group of disorders for which there is no effective therapy, the precise contribution of TGF-β or *Smad4* to fibrotic disease is still unclear [16]. In the case of chronic pancreatitis, progressive fibrosis and destruction of the gland can result in exocrine and endocrine insufficiency. It has been previously shown that loss of TGF-β signaling in fibroblasts results in increased TGFα [18], therefore we set out to examine the possible synergetic effects of *Smad4* loss and TGFα overexpression *in vivo*.

In the present study, we crossed the *MT-TGFα* mouse with a *Smad4* conditional knock-out mouse (*SMAD4* ^*flox/flox*^;*p48-Cre*; hereafter *S4)*, to generate *SMAD4* ^*flox/flox*^;*MT-TGFα;p48-Cre* (hereafter *STP*). We present evidence that more prominent ADM and advanced fibrosis were observed in the *STP* mice than in the *MT-TGFα* mice. PanIN lesions, which were rarely detected in the *MT-TGFα* mice, were a more common occurrence in the *STP* mice. These results demonstrated that although *Smad4* inactivation alone was not sufficient to induce phenotypic changes, it could accerbate the pathological changes initiated by the overexpression of TGFα. Overall, the *STP* mice displayed histologic presentations reminiscent to those of human chronic pancreatitis transitioning to early tumorigenesis. This was confirmed by the upregulated expression of desmoplasia-associated Col1A1 and chronic pancreatitis-related Muc6 detected in the *STP* mice [19], indicating that *STP* mice may be a suitable animal model for studying the transition of chronic pancreatitis to pancreatic cancer.

## Materials and Methods

### Animals and treatments

**Fig 1:**
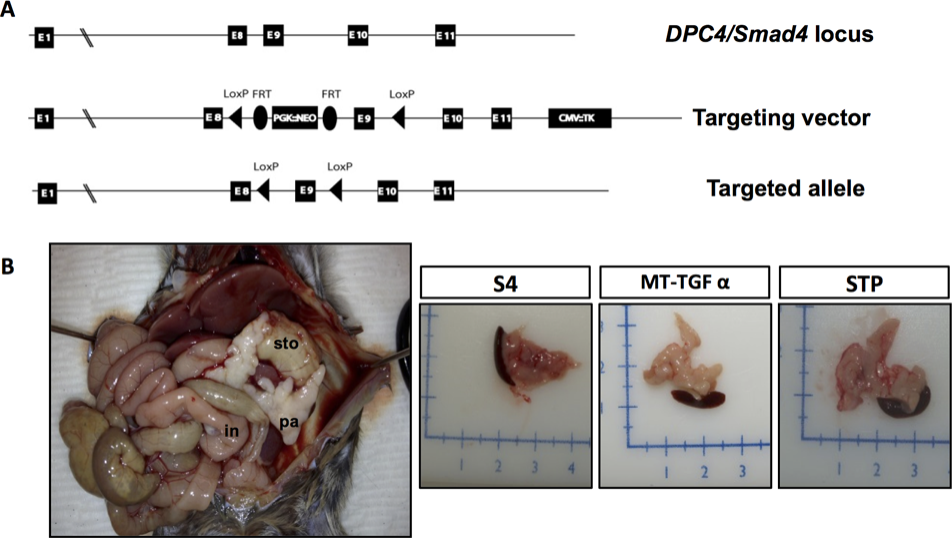
*Smad4* deficiency cooperates with *MT-TGFα* in the growth of fibrotic pancreata. (*A*). Targeting scheme of the *Smad4* ^*flox/flox*^ allele with the introduction of two loxP sites flanking exon 9 of the *DPC4/Smad4* gene. (*B*). Representative *STP* mouse at necropsy (pa: Pancreas; sto: Stomach; in: Intestine) (left). A grossly normal pancreas from a S4 mouse and fibrotic pancreata from *MT-TGFα*, and *STP* mice after 8-months of zinc sulfate treatment (right).

To generate a conditional *Smad4* knockout mouse line (*Smad4* ^*flox/flox*^), exon 9 of the *Smad4* gene was flanked by loxP sites (Fig. 1A). The details on the generation of the *Smad4* ^*flox/flox*^ mouse line are described in the S1 Text. The resulting *Smad4* ^*flox/flox*^ and *Smad4* ^*flox/flox*^;*p48-Cre* mice were live-born and fertile as expected from previous similar publications [13–15]. *MT-TGFα* [9] and *p48-Cre* mice were previously described [20].

*Smad4* ^*flox/flox*^;*MT-TGFα;p48-Cre (STP)*, *Smad4* ^*flox/flox*^;*p48-Cre (S4)*, *and MT-TGFα* mice were treated with zinc sulfate (ZnSO4) water (25mM) for eight months and monitored for significant weight loss, extreme weakness, or inactivity as a sign of illness. At the end of the induction time mice were euthanized and tissues were formalin fixed and paraffin embedded. All studies were conducted in compliance with the guidelines of and with the approval of the Institutional Animal Care and Use Committee (IACUC) of Columbia University Medical Center (Protocol #AC-AAAF1457). Mice were genotyped by Transnetyx Inc. (Cordova, TN).

### Histology and immunohistochemistry

Tissues were fixed in 10% formalin overnight and embedded in paraffin. All pancreata were routinely stained with Hematoxilin & Eosin (H&E) and analyzed by a pathologist (H.E.R.) for any sign of pancreatic tumor development. Stromal proliferation and fibrosis in mice of 8 months of age were qualitatively graded as: mild (1+), defined as surface area of cross section of pancreas composed of <30% stromal; moderate (2+), stromal component comprises 30% to 60% of surface area; strong (3+), stromal component comprises greater than 60% of surface area).

For immunohistochemistry (IHC), slides were deparaffinized in xylene and rehydrated sequentially in ethanol. Antigen retrieval was performed in 0.01M citrate buffer (pH 6.0) for 20 minutes in a microwave oven. Slides were quenched in peroxidase blocking reagent (Dako, Carpinteria, CA) for 30 minutes to block endogenous peroxidase activity. Slides were then incubated overnight at 4°C with primary antibodies diluted in antibody diluent buffer (Dako, Carpinteria, CA). The Dako LSAB-System-HRP kit was employed for signal amplification. Finally, slides were counterstained with hematoxylin, dehydrated sequentially in ethanol, cleared with xylenes, and mounted with Cytoseal 60 (Thermo Fished Scientific, Waltham, MA). The primary antibodies and dilutions were: Smad4, 1:100 (Abcam, Cambridge, MA); CK19, 1:20 (TROMA3, Developmental Studies Hybridoma Bank at the University of Iowa, Iowa City, Iowa); COX-2, 1:100 (cloneSP21, Thermo Scientific, Waltham, MA); Glucagon, 1:1000 (ab932, Millipore, Billerica, MA); Insulin, 1:1000 (A0564, Dako, Carpinteria, CA); α-SMA, 1:200 (ab5694–100, Abcam, Cambridge, MA); Vimentin, 1:350 (ab92547, Abcam, Cambridge, MA), Mucin 6, 1:200 (M-86, Santacruz, Santa Cruz, CA) and p-ERK, 1:500 (#4370, Cell Signaling Technologies, Danvers, MA). Proliferation was assessed by IHC against Ki-67 (clone TEC-3, Dako, Carpinteria, CA). All the antibodies were of rabbit origin except for CK19 (Rat), for which 10 minutes incubation with polyclonal rabbit anti-rat immunoglobulins (E0468, Dako, Carpinteria, CA) preceded the signal amplification step.

For double staining, the primary antibodies anti-amylase (1:2500) (Calbiochem, San Diego, CA) and anti-CK19 (1:50) (TROMA3, Developmental Studies Hybridoma Bank at the University of Iowa, Iowa City, Iowa) were mixed together and the slides were incubated overnight at room temperature. The slides were then incubated with biotinylated anti-rat secondary antibody for 30 min, followed by incubation with Dako Avidin-HRP for another 30 min. DAB substrate (brown) was used for CK19 staining. The ImmPRESS-AP Polymer Anti-Rabbit IgG (Vector Labs, MP-5401) was used for labeling the amylase antibody. The red substrate for alkaline phosphatase (Vector Labs, SK-5100) was used for staining amylase protein. Nuclei were stained with hematoxylin. All the procedures were performed according to the manufacturer’s instructions. Fibrosis was analyzed by Trichrome Masson Staining (Sigma-Aldrich, St. Louis, MO) and Sirius Red (Direct Red 80, Sigma-Aldrich, St. Louis, MO) following the manufacturer’s protocols. For Alcian blue staining, rehydrated paraffin sections were stained for 15 minutes at room temperature in a 3% solution of Alcian blue diluted in acetic acid.

Quantitative analysis of stromal proliferation and fibrosis was achieved by measuring the areas positive for vimentin immunoreaction. Briefly, six random, non-overlapping, 200X images were collected from 4 mice per genotype. For each image positive vimentin area was normalized to total area of each field using ImageJ 1.48g software (NIH, USA). Similar approach was carried out to quantify the area occupied by mucinous lesions. Error bars represent means ± standard error of the mean (S.E.M.). PanIN lesions were classified according to histopathologic criteria recommended in the literature [20, 21].

Images were captured with a Nikon Labophot-2 Microscope using the Nikon Imaging Software NIS-Elements-F 2.20 (Melville, NY).

### Immunoblot analysis

Pancreatic frozen tissues were homogenized using a sonicator in ice-cold RIPA buffer including protease/phosphatase inhibitors (Roche Diagnosis, Indianapolis, IN). Samples were pre-boiled for 5 min and diluted to the same concentration using Laemmli buffer 2x and then separated using Novex 4–20% Tris-Glycine Gels (Invitrogen, Carlsbad, CA). Transfers were done onto a PVDF membrane (Millipore, Temecula, CA). Membranes were pre-blocked with TBST—5% Non-fat dry milk and then incubated with primary antibodies: Vimentin (ab92547, Abcam, Cambridge, MA), Col1a1 (sc-8784-R, Santa Cruz Biotechnology, Santa Cruz, CA) and Beta-actin (sc-47778, Santa Cruz Biotechnology, Santa Cruz, CA). After washing with TBS-0.1% tween-20, secondary antibodies (Cell signaling, Danvers, MA) were added and membranes incubated for 1 hour.

### Expression analysis

Tissues were preserved in RNAlater (Qiagen, Valencia, CA) at -20°C until RNA isolation. RNA was isolated by RNeasy (Qiagen, Valencia, CA) purification and DNAse treatment, followed by reverse transcription (SuperScript III Reverse transcriptase kit, Invitrogen; Carlsbad, CA) and quantitative real-time PCR (Q-PCR), performed in ABI Prism 7500 Sequence Detector (Applied Biosystems, Foster City, CA) using Power SYBR Green PCR Master Mix (Applied Biosystems, Foster City, CA) and the following oligonucleotides: *Col1a1 (Fwd 5’-* ACCTCAAGATGTGCCACTC-3’; Rvs 5’-TGCTCTCTCCAAACCAGAC-3’), *α-SMA (Fwd 5’-* GACGCTGCTCCAGCTATGT*-3’; Rvs 5’-* AGTTGGTGATGATGCCGTGT*-3’)*, *Ctgf (Fwd 5’-* TGACTGCCCCTTCCCGAGAA-3’; Rvs 5’- TCTTCCAGTCGGTAGGCAGCTAGG- 3’), *muc4 (Fwd* 5’- CTCCAAGAAATGTAGTGGCTTTCAG-3’; Rvs 5’- CACGGTCTTGGGCTGGAGTA-3’) *Muc6 (Fwd 5’-*GCCGAGCGTAAATGCAACAT-3’; Rvs 5’- CTCCAAGAAATGTAGTGGCTTTCAG-3’) *Sonic Hedgehog (Fwd 5’-* ACCGAGGGCTGGGATGAGGA-3’; Rvs 5’- ATTTGGCCGCCACGGAGTT-3’). Optimal primer concentrations were determined using optimization protocols from Applied Biosystems SYBR Green PCR Master Mix manual. Relative Quantification was performed and the expression levels were normalized to *Rpl0* with the fold-change calculated based on the ΔΔCt method relative to *S4 mice*.

### Statistical analysis

Results were presented as the mean ± SEM. The Student’s t-test was used to compare data between groups. *p*-values less than 0.05 were considered to be statistically significant.

## Results

### Smad4 is efficiently deleted in STP and S4 mice

**Fig 2:**
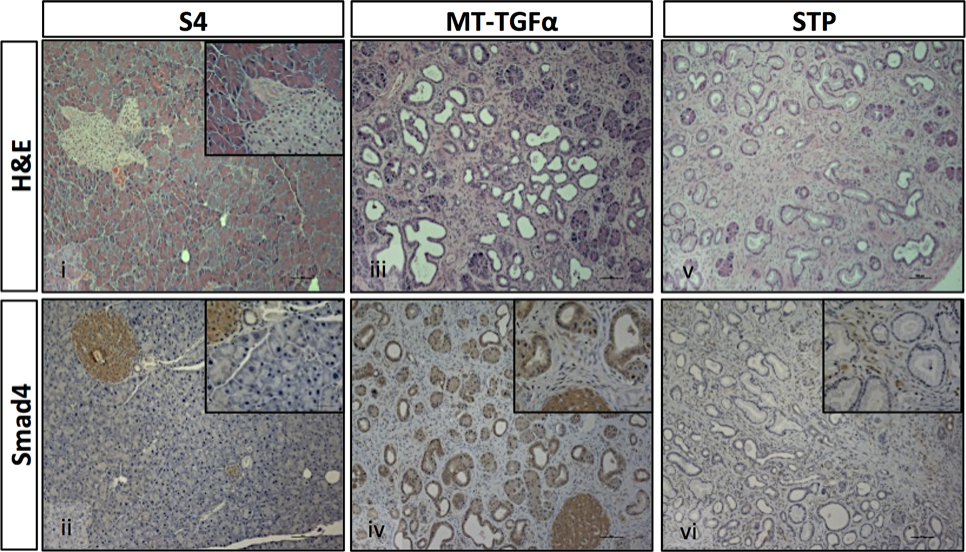
*Smad4* deficiency enhances *TGFα*-induced histological changes. The *STP* mice displayed similar but more pronounced architectural changes in the pancreata than the *MT-TGFα* mice, while the no apparent change was detected in the pancreata of S4 mice. The loss of Smad4 expression in the ductal epithelial cells was confirmed in the *S4* and *STP* mice by IHC. Histological sections from 8-months of zinc sulfate treated *S4* (panels *i*, *ii*), *MT-TGFα* (panels *iii*, *iv*) and *STP* (panels *v*, *vi*) mice stained with H&E (panels *i*, *iii*, *v*); with antibodies to Smad4 (panels *ii*, *iv*, *vi*). Magnifications: panels 100x/200x; insets 400x.

While the *S4* mice presented with normal pancreata, gross inspection of the *STP* mice at necropsy revealed enlarged fibrotic pancreata with frequent cystic formation due to duct dilation similar to those found in the *MT-TGFα* mice [9, 10] (Fig. 1B). Body weight, pancreas to body weight ratio, and survival were similar between the *STP* and *MT-TGFα* mice (data not shown). Tissue-specific Cre-mediated rearrangement of the *Smad4* ^*lox*^ allele in the *S4* and *STP* mice was documented by allele-specific PCR genotyping (S1 Fig.) and further confirmed by Smad4 IHC, demonstrating the generation of a null allele in the pancreas of the *STP* and *S4* mice but not in the *MT-TGFα* mice (Fig. 2). *STP* and *S4* mice presented loss of Smad4 expression in the ductal epithelium while positive expression was detected in the stroma (Fig. 2*ii*, *vi*), whereas *MT-TGFα* mice maintained Smad4 expression in both ductal epithelium and stromal component (Fig. 2*iv*).

### *Smad4* deficiency in the pancreas cooperated with *TGFα* in promoting the expansion of the epithelial compartment and ADM to PanIN progression.

**Fig 3:**
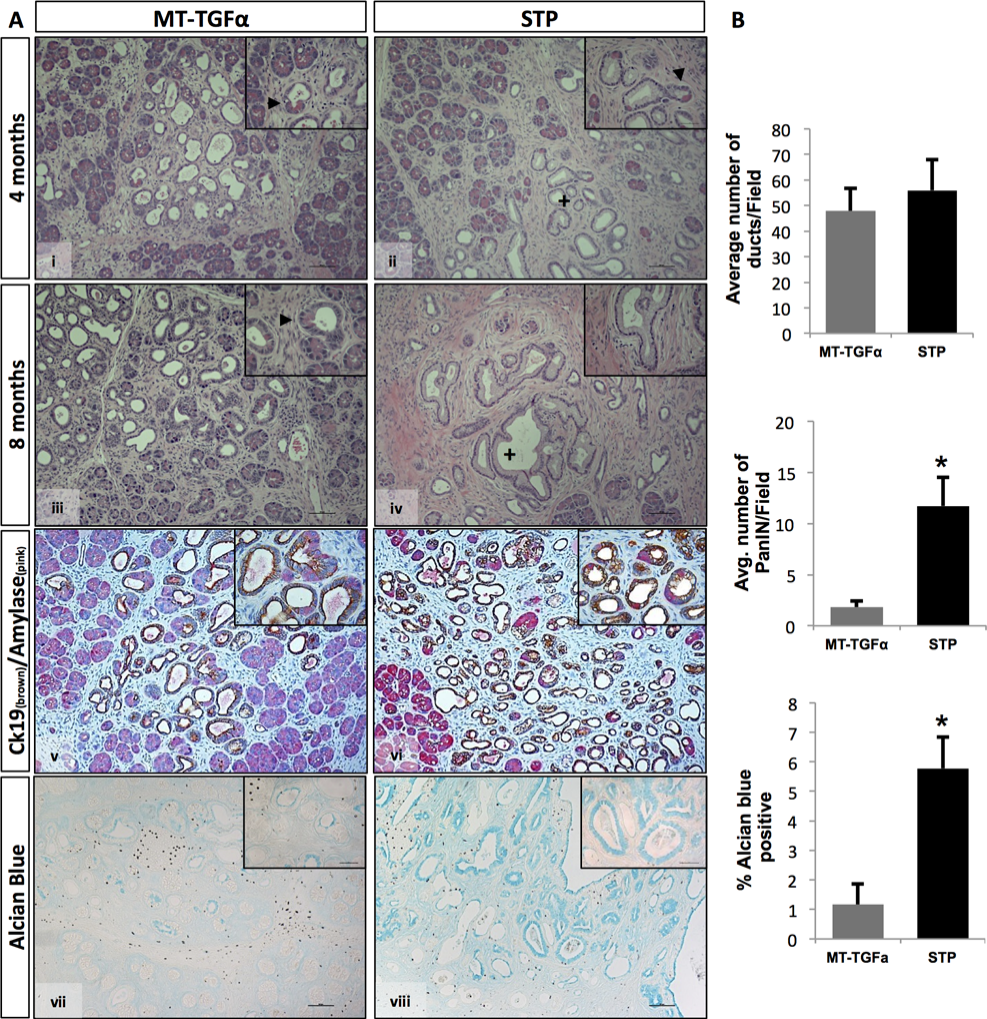
Significant increased number of PanIN lesions observed in the *STP* mice. (*A*) Pancreas specimens from 4 and 8-months zinc sulfate treated *MT-TGFα* (panels *i*, *iii*, *v*, *vii*) and *STP* (panels *ii*, *iv*, *vi*, *viii*) were H&E stained (panels *i-iv*), co-immunolabeled (co-IHC) with antibodies against cytokeratin-19 (brown) and amylase (pink) (panels *v*, *vi*), or Alcian blue stained (*panels vii*, *viii*). *MT-TGFα* mice display ductal proliferation with increased ADM at both 4 and 8 months of treatment (panels *i*, *iii*). *STP* mice showed progression from ADM to PanIN-1 & -2 lesions (panels *ii*, *iv*). Arrows indicate ADM lesions and plus signs denote PanIN-1 lesions. The occurrence of ADMs in both models was demonstrated by co-IHC of Ck19 and amylase (panels *v*, *vi*). Alcian blue indicated vastly increased mucin contents within ducts in the *STP* mice (panel *viii*) over the *MT-TGFα* mice (panel *vii*). (*B*) Morphometric analysis of pancreata from *MT-TGFα* and *STP* mice treated with 8-months of zinc sulfate. Four mice per genotype were analyzed with a minimum of five fields counted per mouse (100x). *(Upper panel)*: The graph depicts the average number of ducts per 200x field. There was no significant difference in the number of total ductal structures counted in both models. (Middle *panel*): The graph depicts the average number of PanIN-1 & -2 lesions per 200x field. The STP mice exhibited marked increase of PanIN-1 & -2 lesions than the *MT-TGFα* mice. (*Lower panel*): Percentage of Alcian blue positive area per 100x field was presented. The increased PanIN lesions were consistent to the enhanced Alcian blue positivity in the *STP* mice. Values are presented as mean ±SEM. (Student’s t-test, ** p < 0.05). Magnifications: panels 100X; insets 400x.

Mice with homozygous deletion of *Smad4* in the pancreas showed no evidence of any gross anatomic or physiological abnormalities, and exhibited normal pancreatic cytoarchitecture (Fig. 1B, Fig. 2*i*, & S2 Fig.). As previously reported, pancreata of the *MT-TGFα* mice underwent a progressive histologic transformation, characterized by diffuse fibrosis and altered acinar cell structure, ADM, ductal proliferation and dilatation [9, 10] (Fig. 2*iii* & Fig. 3A-*i*, *iii*). The *STP* mice showed more pronounced interlobular fibrosis, tubular metaplasia, islands of proliferating cells within the tubules and ADM that involved progressive dilatation of the acinar lumen (Fig. 2*v* & Fig. 3A-*ii*, *iv*). With the decrease in the height of acinar cells, we also observed a cuboidal epithelium morphologically simulating metaplastic ducts and/or PanIN-1 in the *STP* mice (Fig. 3A-*iv*). Both the *MT-TGFα* mice and *STP* mice presented disruption of islet cells due to ductal proliferation and increased fibrosis as shown (S2*v-vii* Fig., S2*ix-xi* Fig.). The diminished exocrine compartment observed was likely due to ADM as demonstrated by co-IHC of amylase and CK19 (Fig. 3A-*v*, *vi* & S2*iv*, *iii*, *xii* Fig.).

**Fig 4:**
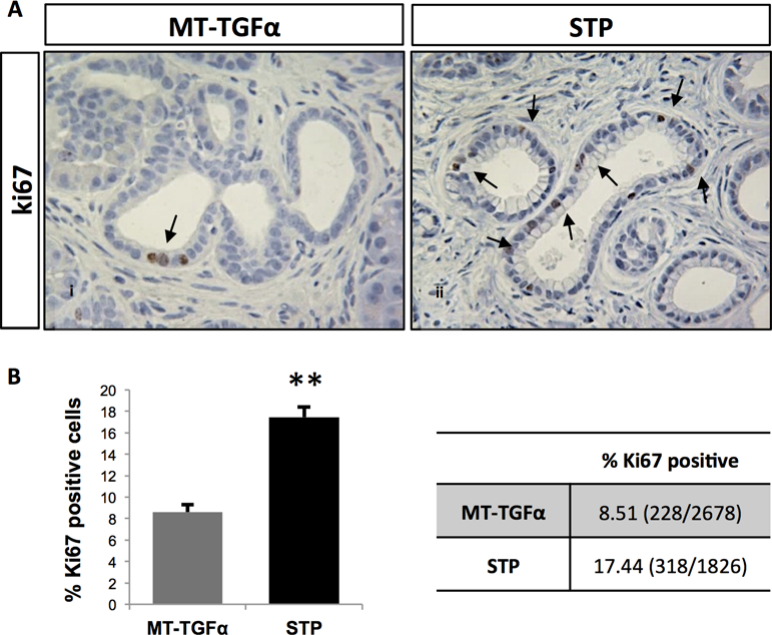
*Smad4* deficiency significantly enhanced *MT-TGFα*-induced epithelial expansion. Increased number of proliferating ductal cells was observed in the *STP* mice over the *MT-TGFα* mice. (*A*) Ki-67 immunolabeling of pancreata from *MT-TGFα* (panel *i*) and *STP* (panel *ii*) mice after 8-months of zinc sulfate treatment. Arrows denote proliferating epithelial cells in evolving ADM and PanIN lesions. (*B*) The graph presents the percentage of Ki-67-positive epithelial cells among the total number of metaplastic cells counted. The table shows the percentage of Ki67-positve cells and in parentheses the total counts of Ki67-positive cells over the total number of metaplastic cells counted from at least three independent mice per genotype and ten 400X fields per mouse are shown. Values are presented as mean ±SE; Magnifications panels *i* and *ii*, 400x. (Student’s t-test, * p < 0.05; ** p < 0.01)

Malignant pancreatic tumors were not detected in any of the mice subjected to zinc sulfate water treatment for up to 8 months. Both the *MT-TGF-α* and *STP* models showed increased numbers of ductal structures (include both metaplastic ducts and/or PanIN-1 & -2) in comparison to the *S4* mice, but with no statistical difference between each other (Fig. 3B). Strikingly, the number of PanIN-1 & -2 lesions was significantly higher in the *STP* mice compared to the *MT-TGFα* mice (Fig. 3B; *MT-TGFα*: 1.8±0.6; *STP*: 11.7±2.8; Student’s t-test, *p* = 0.025). This was supported by the increased positivity for Alcian blue, which is a marker of intestinal mucin, in the *STP* mice when compared to the *MT-TGFα* mice (Fig. 3A; *vii-viii* and Fig. 3B; *MT-TGFα*: 1.2%±0.7; *STP*: 5.8%±0.7; *Student’s t-test*, *p* = 0.016). Analysis of proliferation within the metaplastic ductal epithelia in both models showed a significant increase in the fraction of ki67-positive cells in the *STP* mice when compared to the *MT-TGFα* mice (Fig. 4; *MT-TGFα*: 8.5%±0.7; *STP*: 17.4%±1.0*TGFα*; Student’s t-test, *p* = 0.003). The pronounced epithelial reprogramming resulted from ADM (Fig. 3A-*v*, *vi*) and the heightened ductal proliferation (Fig. 4) likely contributed to the marked increase in the numbers of PanIN lesions in the *STP* mice.

### *Smad4* deficiency enhances *TGFα*-induced fibrosis

**Fig 5:**
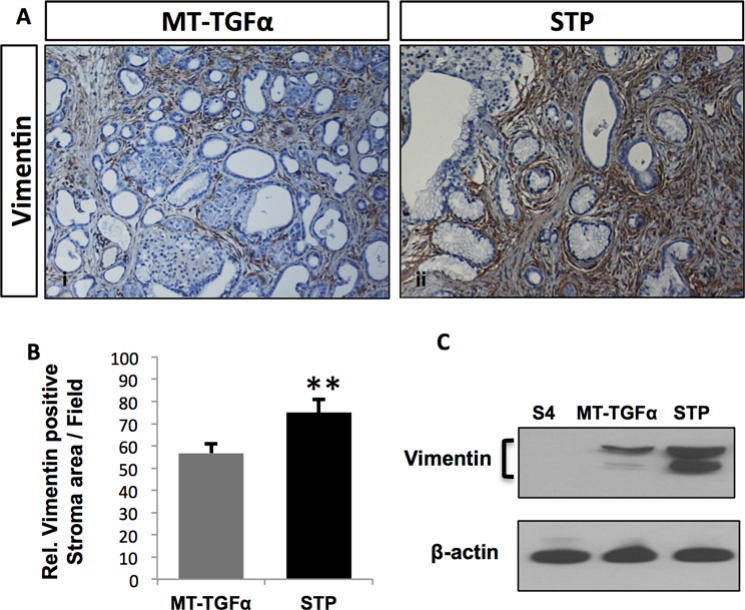
*Aggravated* pancreatic fibrosis promoted by *Smad4* deficiency in the context of TGFα overexpression. Enhanced fibrosis was observed in the *STP* mice as measured by vimentin expression. (*A*) Pancreas specimens from *MT-TGFα* (panel *i)* and *STP* (panel *ii*) treated with 8-months of zinc sulfate were immunolabled with anti-vimentin antibody. Magnifications: panels 100x. (*B*) Morphometric analysis of pancreata from the vimentin IHC. Four mice per genotype were analyzed with six fields counted per mouse. The graph presents the relative stroma area per 200x field based on the vimentin immunoreaction. Values are presented as mean ±SEM. Student’s t-test was employed; * p < 0.05; ** P < 0.01. (*C*) Vimentin protein expressions in the total protein extracts from the pancreata of the *S4*, *MT-TGFα*, and *STP* mice were analyzed by Western blot. β-actin was used as the loading control.

In addition to the changes in the epithelial cells, pancreata of the *STP* mice and the *MT-TGFα* mice displayed progressive accumulation of fibrotic stroma and fibroblasts, demonstrated by increased Trichrome staining, Sirius red staining (S3 Fig.) and vimentin immunoreactivity (Fig. 5A). Qualitative analysis of relative stroma area assessed by a pathologist (H.E.R.) indicated augmented pancreatic fibrosis in the *STP* mice in comparison to the *MT-TGFα* mice, which was confirmed by the quantitative analysis of the relative pancreatic area positive for vimentin expressions by IHC (Fig. 5B; *MT-TGFα*: 56.7%±4.4; *STP*: 75.0%±5.8; Student’s t-test, *p* = 0.003) and also by Western blot analysis of vimentin expressions in the total protein extracts from pancreata of the *S4*, *MT-TGFα* and *STP* mice (Fig. 5C).

Further IHC analysis and expression analysis were performed on the pancreata to interrogate the potential Smad4-dependent molecular alterations in the neoplastic epithelium and in the stroma compartment of the *STP* mice compared to the *MT-TGFα* mice. Activated pancreatic fibroblasts or stellate cells (PSCs) were detected in both models, exclusively in the areas surrounding the ductal lesions (surrounding the ADM in the *MT-TGFα* mice and the ADM and PanINs in the *STP* mice) by *α-*SMA labeling. PSCs are activated through paracrine signals from neighboring cells (acinar, ductal, endothelial and lymphocytes) and/or autocrine signals upon exposure of the pancreas to ethanol, to its metabolites and to insults that generate reactive oxygen species (ROS). Persistent activation of the PSCs is known to make an important contribution to ECM modulation and fibrogenesis [22] (S4A-*i*, *iv* Fig.). RNA expression analysis of *α-SMA* showed higher levels in the *MT-TGFα* and *STP* mice than the *S4* mice, although with similar levels in both models (S4B Fig.; *MT-TGF-α*: 180.9±1.2; *STP*: 198.3± 5.9 Student’s t-test, *p* = 0.0222 and 0.0209 respectively), which was consistent with the observed PSCs in both models. Shh is known to contribute to the formation of desmoplasia in pancreatic cancer [23]. Here Shh was frequently expressed in PanIN lesions of the *STP* mice, and also detected in the ADMs of both the *STP* and *MT-TGFα* mice (S4A-*ii*, *iv* Fig.). *Shh1* RNA expression was upregulated in the *STP* mice compared to the *MT-TGFα* mice, although not significantly (Fig. 4B), probably because upregulated Shh1 expression was detected in both ADM and PanINs (S4A-*ii*, *iv* Fig.). Both the *STP* and *MT-TGFα* models presented significantly higher levels of *Shh1* expressions than the *S4* mice (S4B Fig.; *MT-TGFα*: 1222.8±601.2; *STP*: 3170.2±347.1; Student’s t-test, *p* = 0.0209 and 0.0222 respectively). COX-2 has a cell intrinsic role in pancreatic cancer development and is also required for cancer stimulated PSC proliferation [24, 25] and its heightened expressions in the ADM lesions of both models and in the PanIN lesions and stroma of the *STP* mice were consistent with the observed PSC (S4A Fig.). Both the *STP* and *MT-TGFα* mice showed absent and/or weak labeling for Muc4 and Muc5A; these two mucins are often associated with PDAC development in humans [26–29] (data not shown).

### *Smad4* deficiency cooperates with *TGFα* in the upregulation of molecular markers associated with pancreatitis and desmoplasia

**Fig 6:**
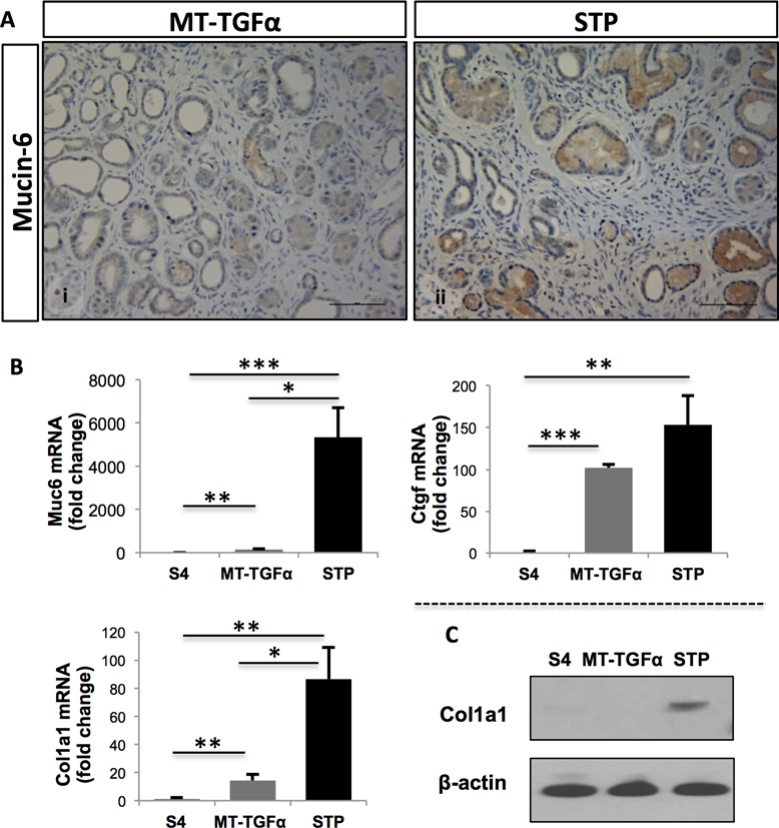
*STP* mice displayed molecular signatures of human chronic pancreatitis and desmoplasia. Significantly elevated Muc6 and Col1a1 expressions were detected in the *STP* mice. (*A*) IHC analysis of Muc6 expression in the pancreata from *MT-TGFα* (panel *i*) and *STP* mice (panel *ii*) treated with zinc sulfate for 8 months. Elevated expression of Muc6 was detected in evolving ADM and PanIN lesions of the *STP* mice. Magnification panels *i* and *ii*, 200X. (*B*) The RNA expressions of chronic pancreatitis marker *Muc 6* and desmoplasia markers *Ctgf* (Connective tissue growth factor-1) and *Col1a1* (Collagen type XXI alpha 1) in the *S4*, *MT-TGFα*, and *STP* mice were measured by qRT-PCR. The graphs present the relative expression of each gene to the *S4* mice. *Muc6* and *Col1a1* were significantly upregulated in the STP mice compared to the *MT-TGFα* mice. Values are presented as mean ±SEM; Student’s t-test was employed, * p < 0.05; **p > 0.01; *** P < 0.005. (*C*) Elevated Col1a1 protein expression in the *STP* mice was confirmed by Western blot analysis of the total protein extracts from the pancreata of the three different models.

The combination of TGFα overexpression and *Smad4* deletion in the pancreas of the *STP* mice presented a number of histologic similarities to human chronic pancreatitis, including increased fibrosis and development of PanIN-1& -2 lesions (S5 Fig.). Therefore, we further assessed the expression of molecular markers that are associated with chronic pancreatitis (Muc6) or desmoplastic reaction (*Ctgf1* and *Col1A1*). Given that mucin genes are highly conserved between human and mice [30] and that *MUC6* has been reported aberrantly expressed in human chronic pancreatitis [19, 27], we sought to analyze Muc6 expression levels in the *STP* and *MT-TGFα* mice. Intriguingly, IHC analysis showed enhanced expression of Muc6 in the lumen of the PanIN lesions in the *STP* mice when compared to the *MT-TGFα* mice (Fig. 6A). Expression analysis of *Muc6* mRNA levels showed that both genotypes overexpressed *Muc6* when compared to *S4* mice. This expression was significantly higher in the *STP* mice than the *MT-TGFα* mice (Fig. 6B; *MT-TGFα*: 146.3±1.3; *STP*: 5354.4±3.2; Student’s test, *p* = 0.0275), indicating, that the *STP* mice resemble not only the fibrotic phenotype (S5 Fig.), but also the overexpression of a molecular marker found in human chronic pancreatitis. Quantitative expression analysis revealed elevated levels of *Ctgf1* in the pancreata of both *STP* and *MT-TGFα* mice when compared to the *S4* mice (Fig. 6B; *MT-TGFα*: 102.3±4.0; *STP*: 153.0±35.3; Student’s t-test, *p* = 0.0059 and 0.0037 respectively), although the differences between the *STP* and *MT-TGFα* mice were not statistically significant (Fig. 6B). Expression analysis of *Col1A1* showed elevated levels in pancreata of both the *STP* and *MT-TGFα* mice when compared to the *S4* mice (Fig. 6B; *MT-TGFα*: 14.3±0.2; *STP*: 86.5±5.8; Student’s t-test, *p* = 0.0056 and 0.0068 respectively). In addition, the *STP* mice also presented significantly higher expression levels of *Col1a1* when compared to the *MT-TGFα* mice (Fig. 6B; Student’s t-test, *p* = 0.010). This was further supported by Western blot analysis (Fig. 6C). Together, our data demonstrate that the *STP* mice present the histologic and molecular signatures that mimic both human chronic pancreatitis and early signs of pancreatic tumorigenesis.

## Discussion

PDAC is the fourth leading cause of cancer death in the United States with a five-year survival as low as 6% [2]. Similar to many other human diseases, mouse models provide an important model system to better understand the molecular mechanism underlying pancreatic cancers. The generation of GEMMs for pancreatic cancer essentially involves targeting a variety of genes in certain pancreatic cell lineages to produce an array of neoplastic changes [31]. Nevertheless, despite the establishment of these GEMMs, no single animal model can provide complete understanding of this disease. Due to the complexity of PDAC biology, novel combinations of models are needed [32].

In the present study, we characterized the *STP mouse* with inducible TGFα overexpression and *Smad4* knock-out in a pancreatic cell lineage-specific manner. As part of the process, we first established the *Smad4* ^*flox/flox*^;*p48-Cre* (*S4*) mouse in which the *Smad4* expression was deleted in the exocrine cells. The pancreas of the *S4* mouse displayed normal histology, which is consistent with previous reports on *Smad4* ^*flox/flox*^;*Pdx1-Cre* mice (Fig. 2) [13–15]. In contrast, the pancreas of a *MT-TGFα mouse* underwent progressive histologic transformation characterized by diffuse fibrosis, tubular metaplasia, ADM, focal PanIN-1 lesions, and islands of proliferating cells within the tubules (Fig. 2). When compared with *MT-TGFα* mice, *STP* mice presented significantly more advanced pancreatic fibrosis, increased foci of metaplastic ductal epithelium, and significantly increased numbers of PanIN lesions (Figs. 2 & 3). In the *STP* mice, there was also evidence of epithelial expansion as indicated by augmented percentage of Ki-67-positive epithelial cells (Fig. 4). However, no pancreatic tumor was detected after prolonged *TGFα* induction beyond one year in both models. The inactivation of *Smad4* likely promoted ADM to PanIN progression in the *STP* mice by enhancing the TGFα signaling. It was recently reported by Chen *et al*. that SMAD4 loss in human pancreatic cancer cell lines led to increased expression of EGFR and the restoration of SMAD4 attenuated EGFR signaling [33]. Consistent to our hypothesis, we detected p-ERK expression in the ADM of the *MT-TGFα* and the *STP* mice (S6*ii-iv* Fig.). Moreover, the pronounced p-ERK expression partially persisted as ADM progressed to PanIN lesions in the *STP* mice (S6 *iii*, *iv* Fig.).

In addition to tumorigenesis, TGF-β is linked to matrix deposition by regulating ECM genes [16]. TGF-β has been shown to contribute to the initiation of fibrotic response *in vivo*. The inactivation of TGF-β signaling by the overexpression of a dominant negative TGF-β receptor II attenuated pancreatic fibrosis and synthesis of ECM proteins induced by repetitive acute pancreatic injuries [17]. While Smad3 expression in fibroblasts has been shown to be essential for the induction of matrix in skin [16, 34], as a downstream mediator of TGF-β signaling, *Smad4* has yet to be assigned a role in pancreatic fibrosis. In our *STP* mice, we demonstrated that the loss of Smad4 expression not only promoted the development of PanIN lesions but also led to aggravated fibrotic changes in comparison to the *MT-TGFα* mice (Fig. 3). The accumulation of fibroblasts in the *STP* mice was demonstrated by increased vimentin expression (Fig. 5). It is worth noting that activated pancreatic stellate cells (PSCs) were detected by α-SMA staining exclusively in areas surrounding the ductal lesions and PanIN lesions in both models (S4 Fig.), which supported the observations of preneoplastic development in this model. This provides the first direct *in vivo* evidence that *Smad4* inactivation in the exocrine lineage would also enhance fibrotic responses (Fig. 3). Fibrosis is generally defined as the accumulation of excessive amounts of ECM proteins in a tissue; in addition to acinar atrophy, fatty replacement, chronic inflammation, and abnormal ducts, pancreatic fibrosis is a constant histopathological hallmark of chronic pancreatitis [35]. In the *MT-TGFα* and *STP* mice, the fibrotic responses were likely initiated by the TGFα expression, which activated the Shh and COX-2 signaling in the stroma (S4 Fig.) and the RAS/MEK/ERK signaling pathway in the ADM of both models and in the PanIN lesions of the *STP* mice (S6 Fig.). The observed interactions between TGFα and TGF-β pathways in promoting pancreatic fibrosis here are supported by previous finding of increased TGFα in fibroblasts losing TGF-β signaling [18], and by the observation of *de novo* loss of heterozygosity in *Smad4* loci in TGFα overexpressing mouse models [36]. The increased fibrotic tissues, activated PSC, and inflammatory pathways observed in the *STP* mice might have resulted directly from the inactivation of *Smad4* or a reactive response to the heightened PanIN development, which cannot be distinguished currently in this study.

Chronic pancreatitis has also been shown to be a risk factor for pancreatic cancer, with the incidence of pancreatic cancer 10 and 20 years after the diagnosis of chronic pancreatitis being 1.8% and 4.0%, respectively [35]. The exact mechanism linking chronic pancreatitis and pancreatic cancer has not been completely defined [5, 37]. While only a small fraction of patients diagnosed with chronic pancreatitis will develop pancreatic cancer, there is no biomarker to predict the cancer development or monitor those at risk. Therefore, novel accurate and sensitive techniques are urgently needed to detect pancreatic cancer at an early stage from chronic pancreatitis [38, 39]. Mucins belong to a family of large glycoproteins that form a physical barrier to protect the epithelial cells from acid, proteases, microorganisms, and mechanical trauma. Alterations in the expression, localization, or glycosylation of mucins have been associated with cancer development, transformation, cancer cell growth, and immune surveillance [40]. While MUC1, MUC4, and MUC5AC are the most differentially overexpressed mucins in human PDAC, the selective increase of MUC6 expression was identified as a potential biomarker for human chronic pancreatitis when compared to normal pancreas and PDAC in an unbiased screen [19]. This finding was corroborated on an independent report studying the expression of mucins in PDAC and chronic pancreatitis [27]. MUC6 has been detected in developing pancreas and in small pancreatic ducts in patients with cystic fibrosis and concomitant chronic pancreatitis, while its role in the pathophysiology of pancreatitis remains unclear [41]. The *STP* mice displayed many of the characteristics described for human chronic pancreatitis (S5 Fig.). The overexpression pattern of Muc6 suggests that our *STP* mice denote some molecular similarity with chronic pancreatitis, in addition to the fibrotic phenotype (Fig. 6). Consistent with the observed PanIN lesions in the *STP* mice, overexpression of *Ctgf and Col1a1*, two biomarkers associated with desmoplasia in human pancreatic cancer were also upregulated in the *STP* mice relative to the *MT-TGFα* mice (Fig. 6B). Of the two markers, *Col1a1* upregulation was statistically significant by qRT-PCR (Fig. 6B), which was further confirmed by Western blot analysis (Fig. 6C). The combination of pancreatitis-like histology and frequent ADM to PanIN progression in the *STP* mice may provide a unique opportunity for interrogating the transition of chronic pancreatitis to pancreatic cancer.

In conclusion, we have established a novel mouse model, the *STP* mice, with inducible TGFα expression and *Smad4* inactivation. The observed advanced ADM, accelerated fibrosis, and increased number of PanIN lesions in the *STP* mice represents a transient state from ADMs to PanINs, which morphologically resembles human chronic pancreatitis associated with PDAC. Compared with the current mouse models used for investigating pancreatic cancers, the *STP* mouse may provide a new opportunity of research in the chronic pancreatitis-PDAC progression.

## Supporting Information

S1 Fig *Smad4* conditional knock-out genotyping and recombination. ***A***, Example of PCR genotyping of the tail DNA of *Smad4* ^*flox/flox*^ and *Smad4* ^*flox/+*^ mice. The primers used for genotyping were: Smad4Gen-F:5’-CCTGTTGTGACGTGGAGG-3’ and Smad4Gen-R:5’-atttgggcagcgtagcaat-3. ***B***, Evidence of Cre-induced recombination in pancreata of *Smad4* ^*flox/flox*^;*p48-Cre*. PCR amplification of genomic DNA yielded a product of 480 bp, the predicted size of the product following recombination at the *loxP* sites flanking the entire exon 9 of the *Smad4* gene. PCR amplification of pancreas DNA from *Smad4* ^*flox/flox*^ littermate failed to yield the recombinant product, indicating no genomic recombination in the absence of the pancreas-specific, *p48-Cre* expression. The primers used for recombination analysis were the following: Smad4Del-F: 5’-ATCGAGGAATTAAGTCATTTTC-3 and Smad4Del-R: 5’-GATAGTTCAGTGATGCCCCT-3’.
(TIFF)

S2 Fig Histological characterization of *STP* mice. Histological sections from the pancreata of *S4* (panels *i-iv*), *MT-TGFα* (panels *v-viii*) and *STP* (panels *ix-xii*) mice after 8-months of zinc sulfate treatment were immunolabled with antibodies to insulin (panels *i*, *v*, *ix*); glucagon (panels *ii*, *vi*, *x*), amylase (panels iii, *viii*, *xi*), and cytokeratin-19 (panels *iv*, *viii*, *xii*). Magnifications: panels 100x; inserts 200x.
(TIFF)

S3 Fig Pronounced pancreatic fibrosis observed in both *MT-TGFα* and *STP* mice. (***A***) Pancreas specimens from *MT-TGFα* (panels *i-ii*) and *STP* (panels *iii-iv*) mice after 8-months of zinc sulfate treatment were stained with Masson’s Trichrome (panels *i*, *iii*) and Sirius Red (panels *ii*, *iv*). Magnifications: 200x.
(TIFF)

S4 Fig Activated pancreatic stellate cells observed in both *STP* mice and *MT-TGFα* mice. (***A***) Pancreatic specimens from 8-months zinc sulfate-treated *MT-TGFα* (panels *i-iii*) and *STP* (panels *iv-vi*) were immunolabeled with antibodies to α-SMA (panels *i*, *iv*), Sonic Hedgehog (panels *ii*, *v*) and COX-2 (panels *iii*, *vi*). The arrows point to positive α-SMA expression in blood vessel in the MT-TGFa mice (panel *i)* and myofibroblast-like cells in the *STP* mice (panel *iv*), and COX-2 labeling in the stroma of *STP* mice (panel *vi*). The asterisks denote Cox-2-positivity in PanIN lesions of the *STP* mice (panel *vi*). (***B***) Relative RNA expressions of αSMA and Sonic Hedgehog in the pancreatic tissues of *S4* (set as 1), *MT-TGFα* (αSMA:180.9±1.2 and Shh:1222.8±601.1) and *STP* (αSMA:198.3±5.9 and Shh:3179.28±347.1) mice as analyzed by qRT-PCR; three mice per genotype. Values are presented as mean ±SEM relative to *S4* mice. Magnifications: panels *i*, *iii*, *iv and vi* 200x; panels *ii*, *v* 100X; insets 400X. (Student’s t-test, * p < 0.05).
(TIFF)

S5 Fig *STP* mice develop features similar to human chronic pancreatitis (*A*). Representative H&E staining of human PC showing increased fibrosis, ductal proliferation, PanIN-1 and destruction of normal pancreatic architecture. (***B***) Representative H&E staining of STP mouse treated with Zinc Sulfate for 8-months showing similar features to human CP. ac: acinar cells; *: Ductal proliferation; +: PanIN-1. Magnification, 100X.
(TIFF)

S6 Fig MAPK activation was detected in the ADM and PanIN lesions in the *STP* mice. Representative pancreatic specimens from 8-months zinc sulfate-treated *S4* (panel *i*), *MT-TGFα* (panel *ii)* and *STP* (panels *iii-iv)* were immunolabeled with antibody to p-ERK. MAPK activation was detected in the normal ducts of the *S4* mice (panel i), ADM of *MT-TGFα* and *STP* mice (panels *ii* and *iii*), and in some PanIN lesions of the *STP* mice (panel *iii*, *iv*). Magnification, 100X for panel iii and 200X for *panels i*, *ii*, & *iv*; insets 400X.
(TIFF)

S1 Text Construction of the conditional Smad4 targeting vector. (DOCX)
