# Supplementary figures and images for: Smad4 Loss Synergizes with TGFα Overexpression in Promoting Pancreatic Metaplasia, PanIN Development, and Fibrosis
Source: PLoS One. 2015 Mar 24;10(3):e0120851. doi: 10.1371/journal.pone.0120851 (PMC4372593; doi:10.1371/journal.pone.0120851)

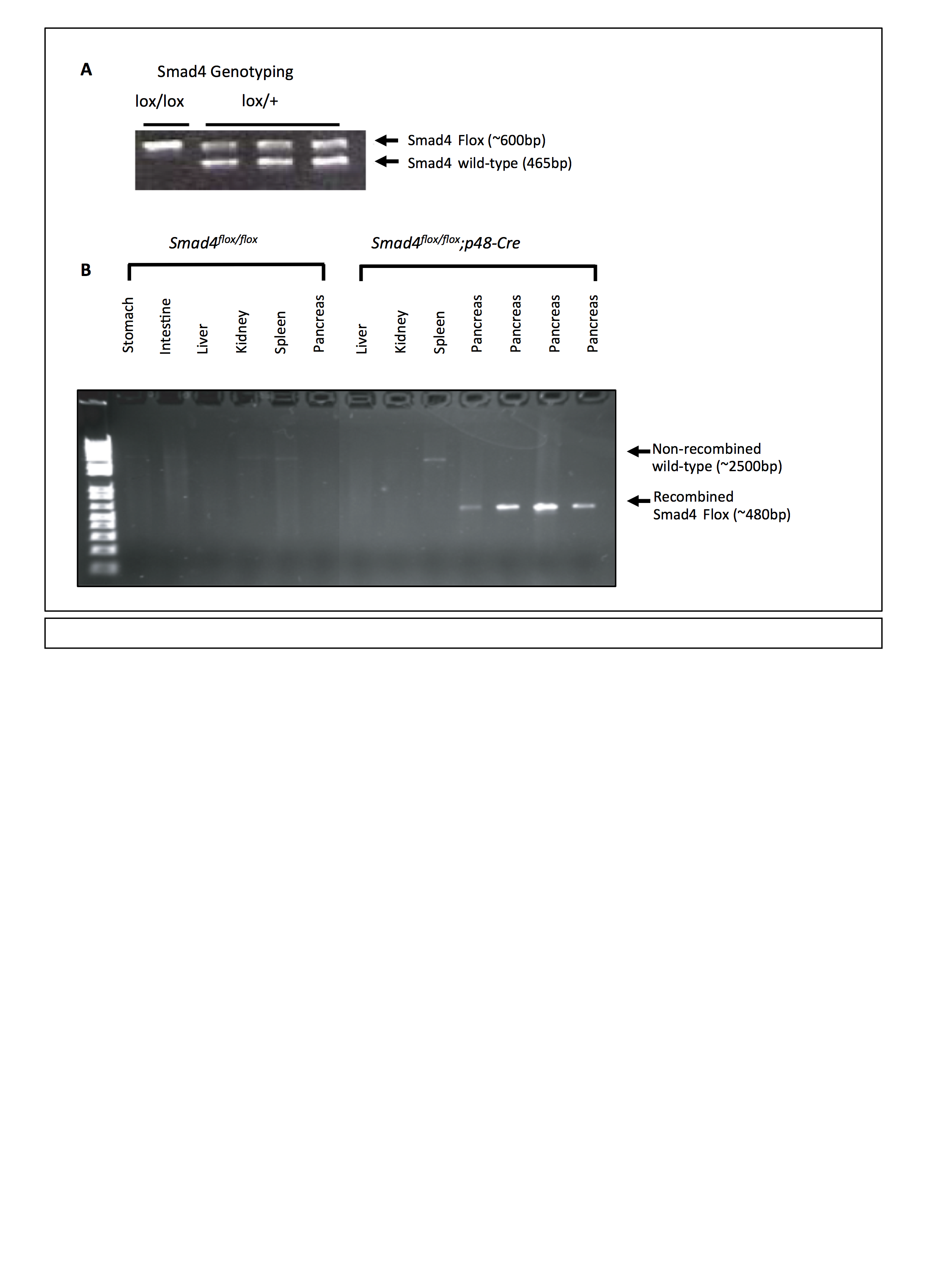

Supplement: S1 Fig — A, Example of PCR genotyping of the tail DNA of Smad4 flox/flox and Smad4 flox/+ mice. The primers used for genotyping were: Smad4Gen-F:5’-CCTGTTGTGACGTGGAGG-3’ and Smad4Gen-R:5’-atttgggcagcgtagcaat-3. B, Evidence of Cre-induced recombination in pancreata of Smad4 flox/flox;p48-Cre. PCR amplification of genomic DNA yielded a product of 480 bp, the predicted size of the product following recombination at the loxP sites flanking the entire exon 9 of the Smad4 gene. PCR amplification of pancreas DNA from Smad4 flox/flox littermate failed to yield the recombinant product, indicating no genomic recombination in the absence of the pancreas-specific, p48-Cre expression. The primers used for recombination analysis were the following: Smad4Del-F: 5’-ATCGAGGAATTAAGTCATTTTC-3 and Smad4Del-R: 5’-GATAGTTCAGTGATGCCCCT-3’. (TIFF) [file pone.0120851.s001.tiff]

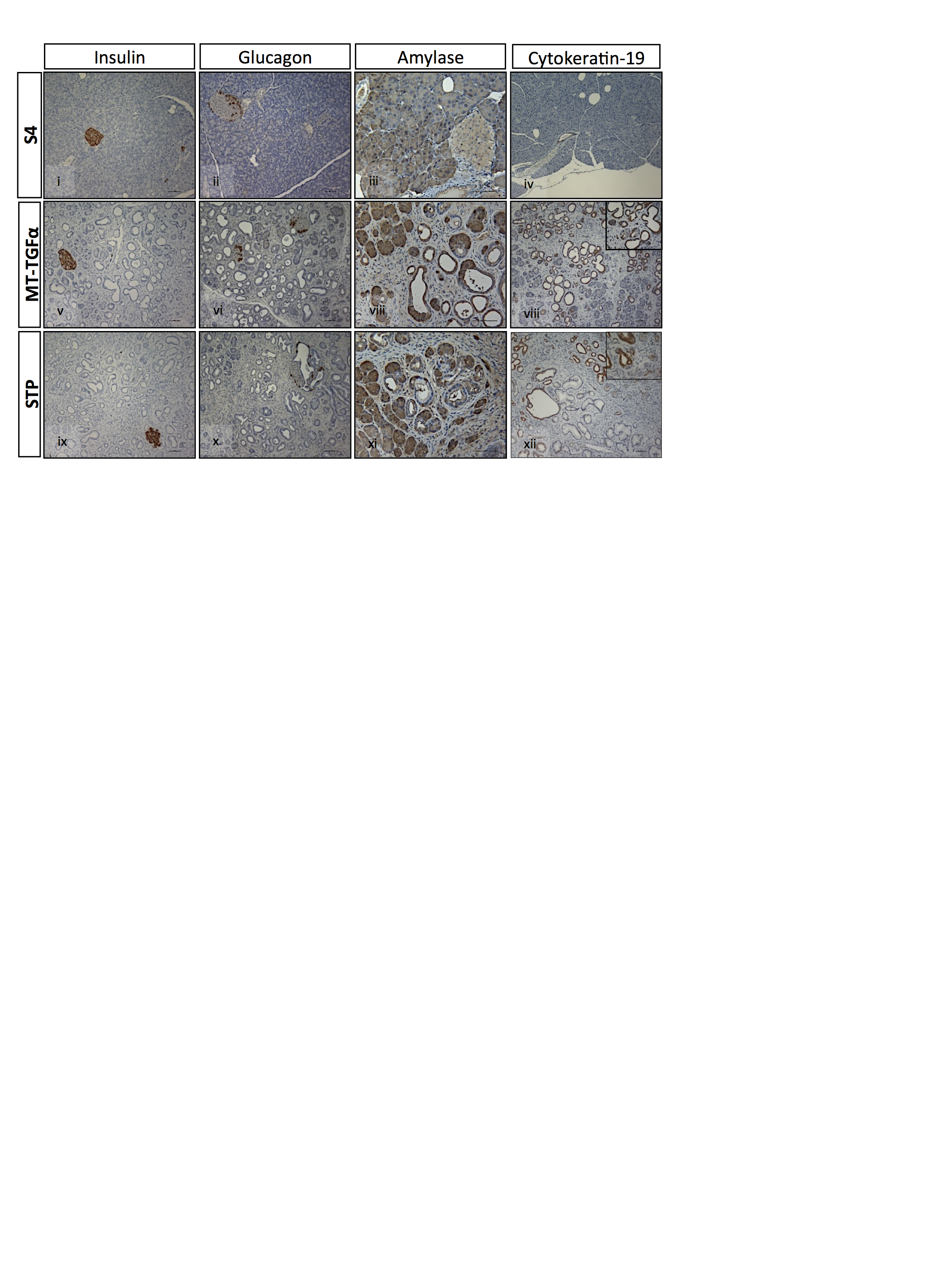

Supplement: S2 Fig — Histological sections from the pancreata of S4 (panels i-iv), MT-TGFα (panels v-viii) and STP (panels ix-xii) mice after 8-months of zinc sulfate treatment were immunolabled with antibodies to insulin (panels i, v, ix); glucagon (panels ii, vi, x), amylase (panels iii, viii, xi), and cytokeratin-19 (panels iv, viii, xii). Magnifications: panels 100x; inserts 200x. (TIFF) [file pone.0120851.s002.tiff]

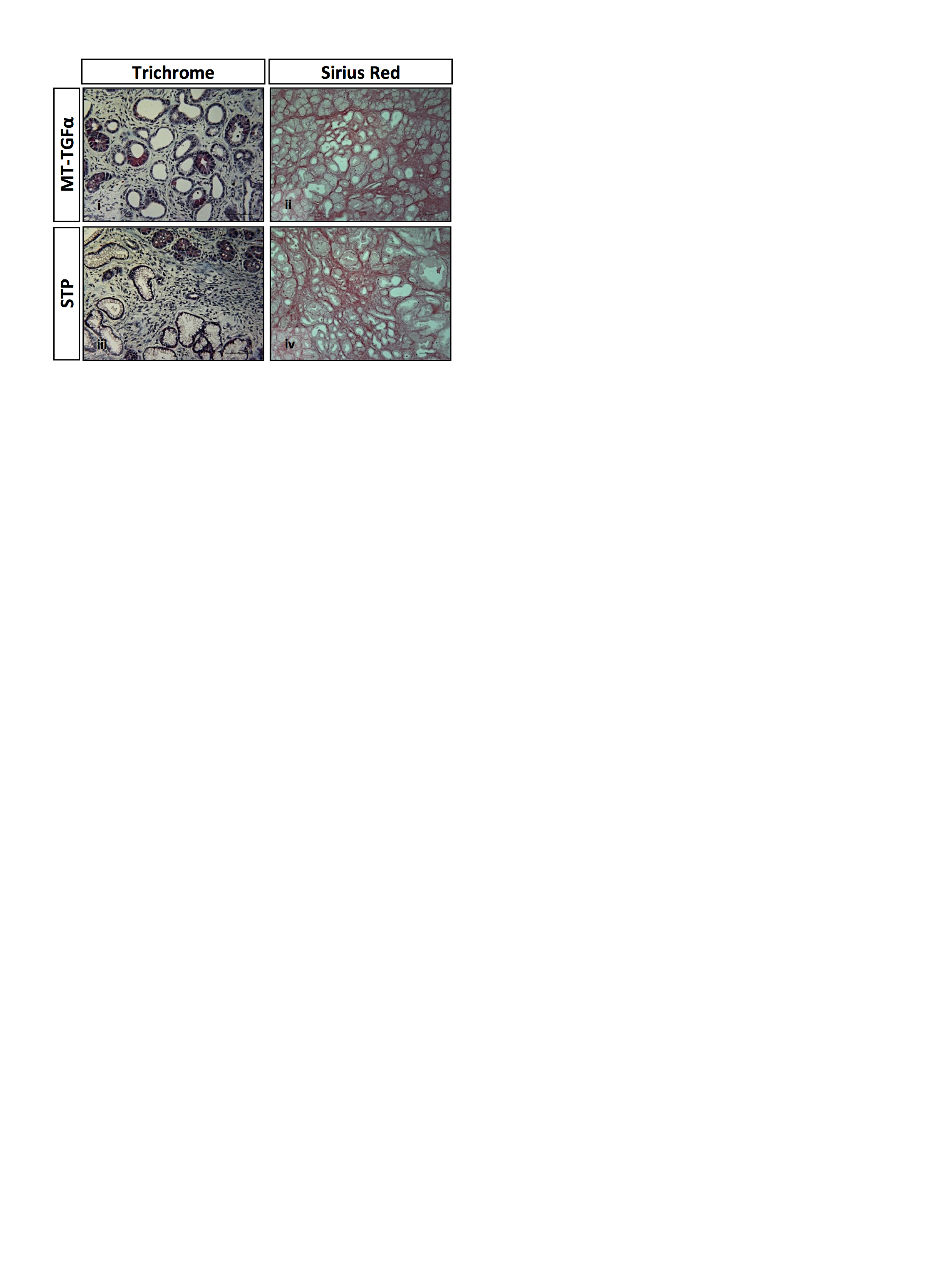

Supplement: S3 Fig — (A) Pancreas specimens from MT-TGFα (panels i-ii) and STP (panels iii-iv) mice after 8-months of zinc sulfate treatment were stained with Masson’s Trichrome (panels i, iii) and Sirius Red (panels ii, iv). Magnifications: 200x. (TIFF) [file pone.0120851.s003.tiff]

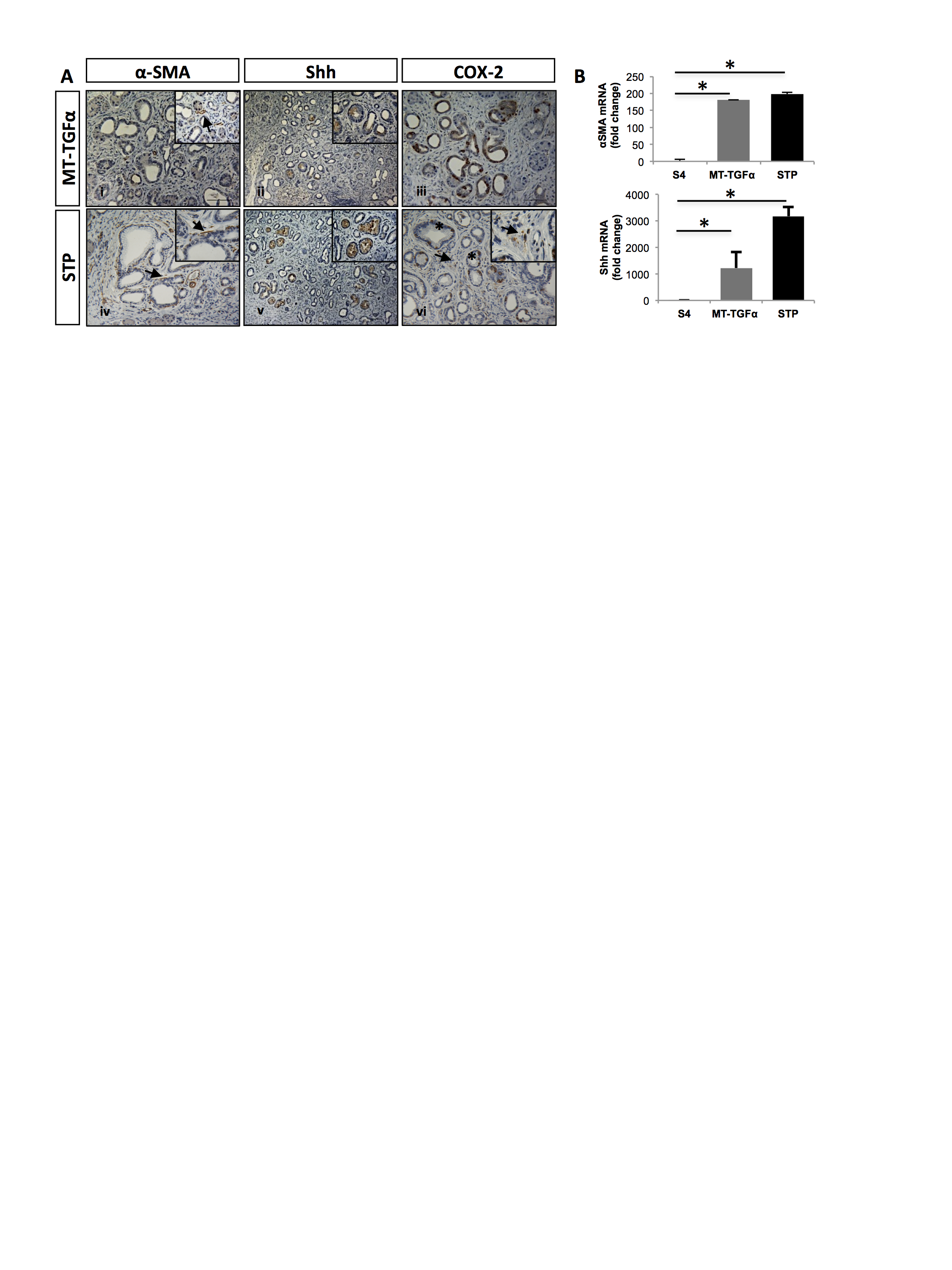

Supplement: S4 Fig — (A) Pancreatic specimens from 8-months zinc sulfate-treated MT-TGFα (panels i-iii) and STP (panels iv-vi) were immunolabeled with antibodies to α-SMA (panels i, iv), Sonic Hedgehog (panels ii, v) and COX-2 (panels iii, vi). The arrows point to positive α-SMA expression in blood vessel in the MT-TGFa mice (panel i) and myofibroblast-like cells in the STP mice (panel iv), and COX-2 labeling in the stroma of STP mice (panel vi). The asterisks denote Cox-2-positivity in PanIN lesions of the STP mice (panel vi). (B) Relative RNA expressions of αSMA and Sonic Hedgehog in the pancreatic tissues of S4 (set as 1), MT-TGFα (αSMA:180.9±1.2 and Shh:1222.8±601.1) and STP (αSMA:198.3±5.9 and Shh:3179.28±347.1) mice as analyzed by qRT-PCR; three mice per genotype. Values are presented as mean ±SEM relative to S4 mice. Magnifications: panels i, iii, iv and vi 200x; panels ii, v 100X; insets 400X. (Student’s t-test, * p < 0.05). (TIFF) [file pone.0120851.s004.tiff]

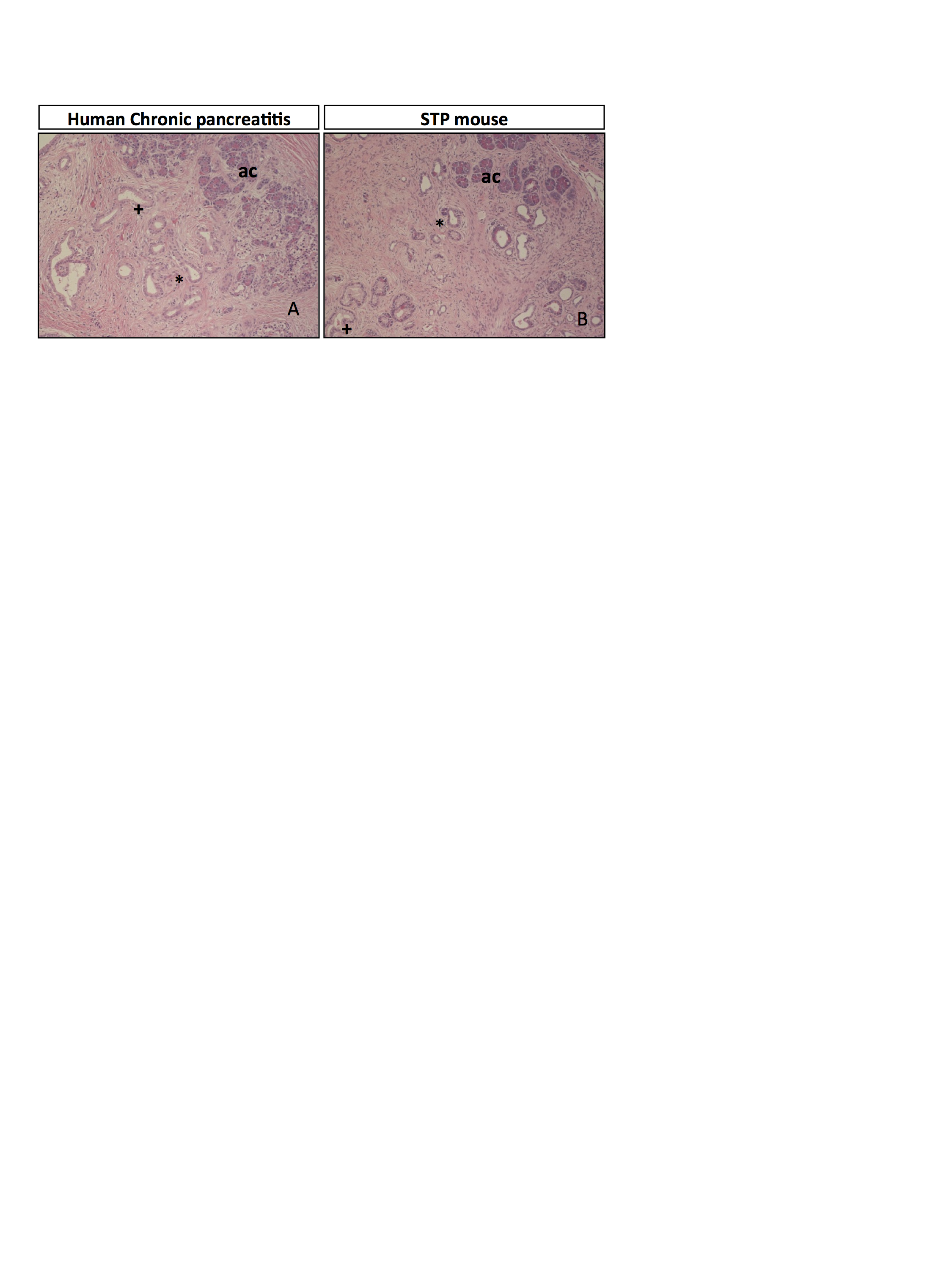

Supplement: S5 Fig — Representative H&E staining of human PC showing increased fibrosis, ductal proliferation, PanIN-1 and destruction of normal pancreatic architecture. (B) Representative H&E staining of STP mouse treated with Zinc Sulfate for 8-months showing similar features to human CP. ac: acinar cells; *: Ductal proliferation; +: PanIN-1. Magnification, 100X. (TIFF) [file pone.0120851.s005.tiff]

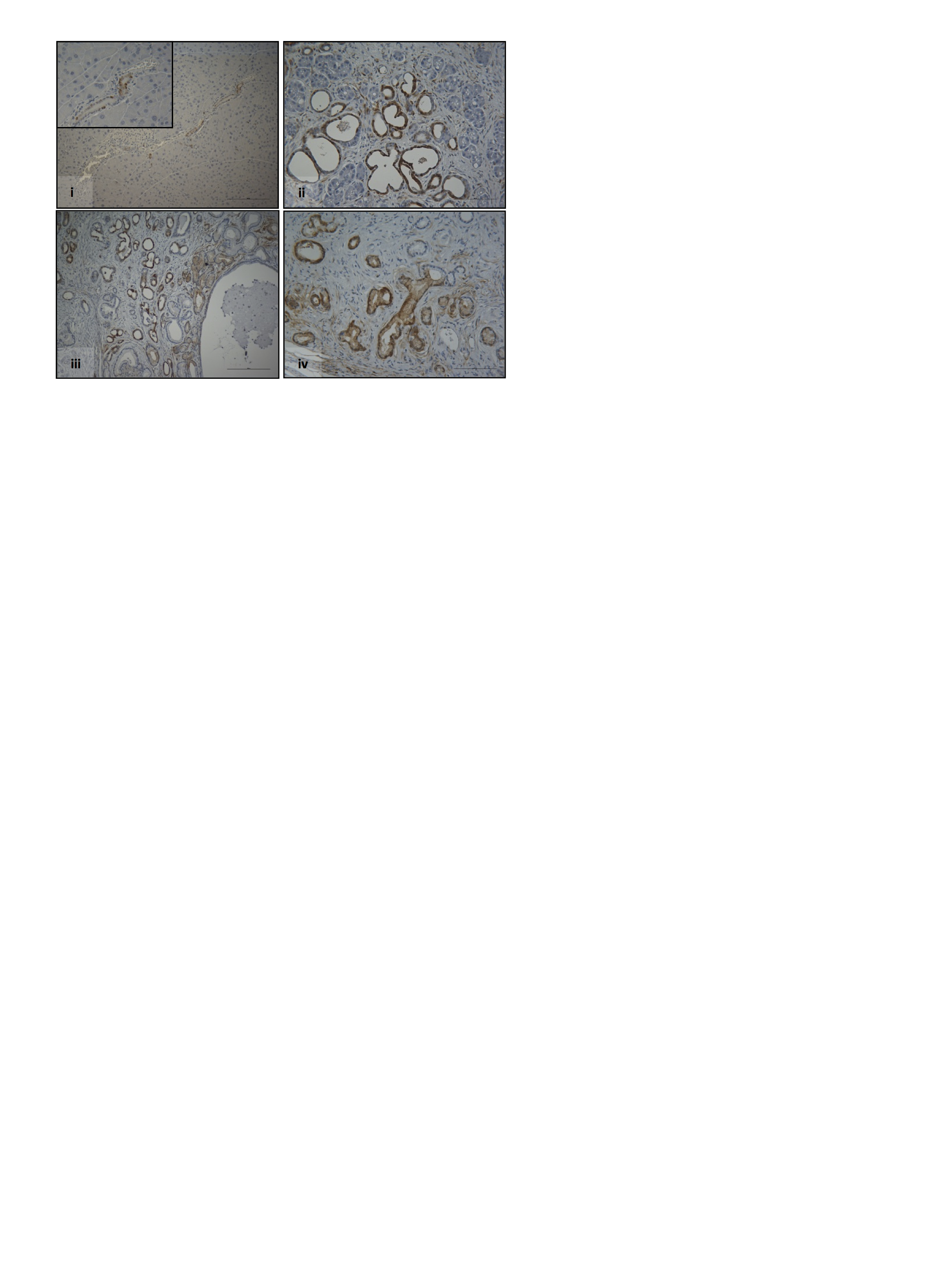

Supplement: S6 Fig — Representative pancreatic specimens from 8-months zinc sulfate-treated S4 (panel i), MT-TGFα (panel ii) and STP (panels iii-iv) were immunolabeled with antibody to p-ERK. MAPK activation was detected in the normal ducts of the S4 mice (panel i), ADM of MT-TGFα and STP mice (panels ii and iii), and in some PanIN lesions of the STP mice (panel iii, iv). Magnification, 100X for panel iii and 200X for panels i, ii, & iv; insets 400X. (TIFF) [file pone.0120851.s006.tiff]
