# Supplementary material for: Smad4 Loss Synergizes with TGFα Overexpression in Promoting Pancreatic Metaplasia, PanIN Development, and Fibrosis
Source: PLoS One. 2015 Mar 24;10(3):e0120851. doi: 10.1371/journal.pone.0120851 (PMC4372593; doi:10.1371/journal.pone.0120851)
Supplement: S1 Text — (DOCX) [file pone.0120851.s007.docx]

**S1 Text. Construction of the conditional Smad4 targeting vector.**

The transactivation domain of *Smad4*, MH2 domain, is encoded by exons 8-11 in human and mouse. Replacement of exons 8 and 9 with neomycin resistance gene (*NEO*) led to null expression of *Smad4* [[1](#_ENREF_1)]. Adopting this same strategy, we designed our targeting construct to delete exon 9 upon Cre induction (Figure 1). A 129/Sv mouse genomic library (Stratagene; Lambda FIXII) was screened with a mouse *Smad4* cDNA probe containing exons 8, 9, 10 and 11 of the *Smad4* coding region and a 15390-bp lambda clone was acquired to construct the conditional *Smad4* targeting vector (DPC4-lopxP: 21000bp). Short and long arm fragments from the lambda clone were inserted into the pNeo-FRT-LoxP vector (a gift of Drs. Kogo Takamiya and Richard L. Huganir at The Johns Hopkins University) subsequently [[2](#_ENREF_2), [3](#_ENREF_3)]. The construct was designed so that Flp-mediated recombination would remove the Neo-cassette from the targeting allele *in vivo*, and Cre-mediated recombination would lead to the deletion of a ~5kb genomic sequence containing exon 9. This recombination would inactivate *Smad4* expression in a tissue-specific manner (S1 Fig).

**REFERENCES**

1. Sirard C, de la Pompa JL, Elia A, Itie A, Mirtsos C, Cheung A, et al. The tumor suppressor gene Smad4/Dpc4 is required for gastrulation and later for anterior development of the mouse embryo. Genes Dev. 1998;12:107-19.

2. Qiu W, Li X, Tang H, Huang AS, Panteleyev AA, Owens DM, et al. Conditional Activin Receptor Type IB (Acvr1b) Knockout Mice Reveal Hair Loss Abnormality. J Invest Dermatol. 2011;131(5):1067-76. Epub Dec 30, 2010.

3. Qiu W, Sahin F, Iacobuzio-Donahue CA, Garcia-Carracedo D, Wang WM, Kuo CY, et al. Disruption of p16 and Activation of Kras in Pancreas Increases Ductal Adenocarcinoma Formation and Metastasis in vivo. Oncotarget. 2011;2(11):862-73. Epub Nov 2011.
